# Association between ADIPOQ gene variants and knee osteoarthritis in a Chinese population

**DOI:** 10.1042/BSR20182104

**Published:** 2019-03-01

**Authors:** Houlai Shang, Yuedong Hao, Wenhao Hu, Xiaohui Hu, Qing Jin

**Affiliations:** 1 Department of Orthopaedics, The Affiliated Huaian No.1 People’s Hospital of Nanjing Medical University, Huaian 223300, Jiangsu, China; 2 Department of Operation and Anesthesiology, The Affiliated Huaian No.1 People’s Hospital of Nanjing Medical University, Huaian 223300, Jiangsu, China

**Keywords:** ADIPOQ, knee osteoarthritis, single nucleotide polymorphisms

## Abstract

A study from Thailand showed no significant association between the adiponectin (ADIPOQ) gene rs1501299 polymorphism and knee osteoarthritis (OA) risk. To investigate this association in a Chinese population, we conducted this case–control study involving 372 knee OA patients and 453 controls. Genotyping via standard PCR and restriction fragment length polymorphism (PCR-RFLP) showed that TT genotype (TT vs. GG: adjusted odds ratio (OR) (95% confidence interval (CI)) = 1.70 (1.01–2.86)) or T allele (T vs. G: adjusted OR (95% CI) = 1.26 (1.02–1.56)) of ADIPOQ gene rs1501299 polymorphism significantly increased the risk of knee OA. Significant associations were also observed in subgroups ≥55 years (TT vs. GG: adjusted OR (95% CI) = 2.21 (1.00–4.86)) and body mass index (BMI) < 25 kg/m^2^ (TT+GT vs. GG: adjusted OR (95% CI) = 1.53 (1.03–2.29)), but not in the subgroup analysis of sex. In conclusion, the ADIPOQ gene rs1501299 polymorphism intensifies the risk of knee OA in this Chinese Han population. Nevertheless, further studies with larger sample sizes in other populations are warranted to verify this finding.

## Introduction

Osteoarthritis (OA) is a clinical syndrome of joint pain and dysfunction that attacks millions of people [1]. OA is responsible for activity limitations, particularly walking, and affects participation and quality of life [2]. Amongst the weight-bearing joints, the knee is the most frequent-occurring place of OA [3]. However, the etiological factors involved in OA remain unclear. Obesity is reportedly a primary risk factor for knee OA [4–6]. Age, sex, body mass index (BMI), and repetitive joint activity are all involved in OA development [7,8]. In addition to age, excessive BMI, joint surgery, and trauma that are recognized as the causes of OA, genome-wide association studies show genetic factors also contribute to OA development [9–11]. Therefore, environmental factors, genetic factors, and their interactions are associated with OA susceptibility [12,13].

Adipose tissues, an active endocrine organ, release some adipokines that are pivotal in bone formation and bone absorption, including adiponectin (ADIPOQ), leptin, and visfatin [14]. Adipokines can stimulate the secretion of interleukin (IL)-6/8 and prostaglandin E2 in synovial fibroblasts [15–17], and are implicated in the development and progression of OA [18]. ADIPOQ level is associated with radiographic changes and progression of rheumatoid arthritis [19,20], and with cartilage destruction in OA patients [21]. Synovial fluid ADIPOQ level can promote inflammation in OA patients and contribute to OA-related metabolic changes [22]. ADIPOQ is also linked with cartilage loss [23]. Serum ADIPOQ level could be used as a biomarker of OA [24]. A recent meta-analysis shows that ADIPOQ levels are higher in OA patients than in healthy controls [25], which suggests an important role of ADIPOQ in OA development.

Recently, several studies have explored the association between ADIPOQ gene polymorphisms and OA risk [26–28], but present contradictory and inconclusive findings. Thus, this case–control study was conducted to verify such relationship in a Chinese Han population.

## Materials and methods

### Patients

A total of 372 knee OA patients were selected from Huai’an First People’s Hospital from July 2013 to June 2017. According to the criteria of the American College of Rheumatology, primary OA was diagnosed as any symptom and sign of OA as well as radiographic signs of OA based on the Kellgren–Lawrence (K-L) grade [29]. Exclusion criteria were as follows: (i) ankylosing spondylitis, psorasis, and other autoimmune diseases; (ii) previous knee injury or joint infection; (iii) alcohol abuse for more than 6 months. A total of 453 healthy controls without family history of OA were recruited from the patients who attended the general surgery of the same hospital at the same time period. Any individual with a positive history of tumor, other arthritis or joint diseases, or other inflammatory or chronic infectious symptoms was excluded. All subjects were from the same ethnic and geographical origins, and there was no genetic relationship between groups. The clinical characteristics of all OA patients and controls including sex, age, K-L grade, and BMI were extracted from medical records. Informed consents were obtained from all participants before the research. The study protocol was approved by the Institutional Review Board of the Hospital. The present study was carried out in accordance with the World Medical Association Declaration of Helsinki.

### DNA extraction and genotyping

Blood samples (each 2 ml) were collected in vacutainer tubes and transferred to EDTA tubes. Genomic DNA was extracted from the peripheral blood (each sample 300 μl) using a QIAamp DNA blood mini-kit (Qiagen, Hilden, Germany). The selective single-nucleotide polymorphism (SNP) was genotyped via standard PCR and restriction fragment length polymorphism (PCR-RFLP). The following forward and reverse primers were used: 5′-ACACTGATATAAACGCCATGAA­-3′ and 5′-­GCAGCAAAGCCAAAGTCTTC-­3′. In brief, a PCR system (50 μl) consisted of 10× PCR buffer for KOD-Plus-Neo (5 μl), ddH_2_O (34 μl), primers (each 1 μl), template (1 μl), 2 mM dNTPs (5 μl), 25 mM MgSO_4_ (3 μl), cDNA (1 μl), and KOD-Plus-Neo (TOYOBO, Japan, 1 μl). Reaction conditions were: 95°C, 5 min; 94°C, 30 s; 50°C, 30 s; 72°C, 1 min, 35 cycles; 72°C, 10 min for extension, cooling to 4°C. The PCR products were digested with BglI (New England Biolabs, Beverly, MA) at 37°C for 5 h and then detected via 2% agarose gel electrophoresis. Approximately 5% of the samples were randomly chosen for a second run to validate the accuracy of the genotyping results. All duplicate samples showed a concordance rate of 100%.

### ELISA

Plasma ADIPOQ levels were determined with an ADIPOQ (Human) ELISA kit (Shanghai Qunji Biotech Co., Ltd, China) and calculated by referring to a standard curve.

### Statistical analysis

The differences in demographic variables, allele frequencies, and genotypes amongst all individuals were assessed via Chi-squared (χ^2^) test. Odds ratios (ORs) and 95% confidence intervals (CIs) were calculated by logistic regression analysis. The Hardy–Weinberg equilibrium (HWE) amongst controls was examined by a goodness-of- fit χ^2^ test. Plasma ADIPOQ levels for genotypes were analyzed by one-way ANOVA and Student’s *t*test. All statistical analyses were performed on SAS 9.1.3 (SAS Institute, Cary, NC, U.S.A.).

## Results

### Characteristics of the study population

The detailed characteristics of all individuals and the K-L grades of OA cases were summarized in Table 1. The mean ages of the cases and controls were 50.23 and 51.12 year, respectively. The female to male ratio of OA cases was 57.5%:42.5%. The mean BMI of cases was 25.01 kg/m^2^. No significant differences in sex, age, or BMI were found between the OA patients and controls. The OA patients showed significantly higher ADIPOQ levels than the controls (*P*=0.022).

**Table 1:** Subject demographics and risk factors in knee OA

Variables	Patients (*n*=372)	Controls (*n*=453)	*P*
Sex			0.674
Male	158 (42.5%)	199 (43.9%)	
Female	214 (57.5%)	254 (56.1%)	
Age (years)	50.23 ± 10.91	51.12 ± 9.93	0.880
BMI (kg/m^2^)	25.01 ± 1.71	25.04 ± 1.52	0.779
K-L grade			
1	73 (19.6%)	-	-
2	121 (32.5%)	-	-
3	155 (41.7%)	-	-
4	23 (6.2%)	-	-
ADIPOQ levels (μg/ml)	5.53 ± 3.54	3.70 ± 2.36	**0.022**

Values in bold are statistically significant (*P* <0.05). ADIPOQ levels were available in 30 OA patients (TT: 10; TG: 10; GG: 10) and 30 healthy controls (TT: 10; TG: 10; GG: 10).

### Association between ADIPOQ gene rs1501299 polymorphism and knee OA risk

Genotype distributions of ADIPOQ gene rs1501299 polymorphism in the control group conformed to HWE (*P*=0.566; Table 2). Logistic regression analyses showed TT genotype of this polymorphism significantly increased the risk of knee OA (TT vs. GG: adjusted OR and 95% CI, 1.70 (1.01–2.89), *P*=0.046) (Table 2). The allele genetic analysis showed T allele was associated with an increased risk of knee OA (Table 2).

**Table 2:** Logistic regression analysis of associations between rs1501299 polymorphism and risk of knee OA

Genotype	Cases* (*n*=372)		Controls* (*n*=453)		OR (95% CI); *P*	Adjusted OR (95% CI); *P*
	*n*	%	*n*	%		
GT vs. GG	160/174	42.7/46.8	182/240	40.2/53.0	1.21 (0.91, 1.62); 0.190	1.21 (0.91, 1.62); 0.187
TT vs. GG	37/174	9.9/46.8	30/240	6.6/53.0	**1.70 (1.01, 2.86);** 0.045	**1.70 (1.01, 2.89);** 0.046
TT+GT vs. GG	197/174	52.6/46.8	212/240	46.8/53.0	1.28 (0.97, 1.69); 0.077	1.28 (0.97,1.69); 0.076
TT vs. GT+GG	37/334	9.9/89.5	30/422	6.6/93.2	1.56 (0.94, 2.58); 0.084	1.55 (0.94, 2.57); 0.085
T vs. G	234/508	31.3/68.1	242/662	26.7/73.1	**1.26 (1.02, 1.56);** 0.034	

* The genotyping was successful in 371 cases and 452 controls.

Bold values are statistically significant (*P*<0.05).

**Figure 1:**
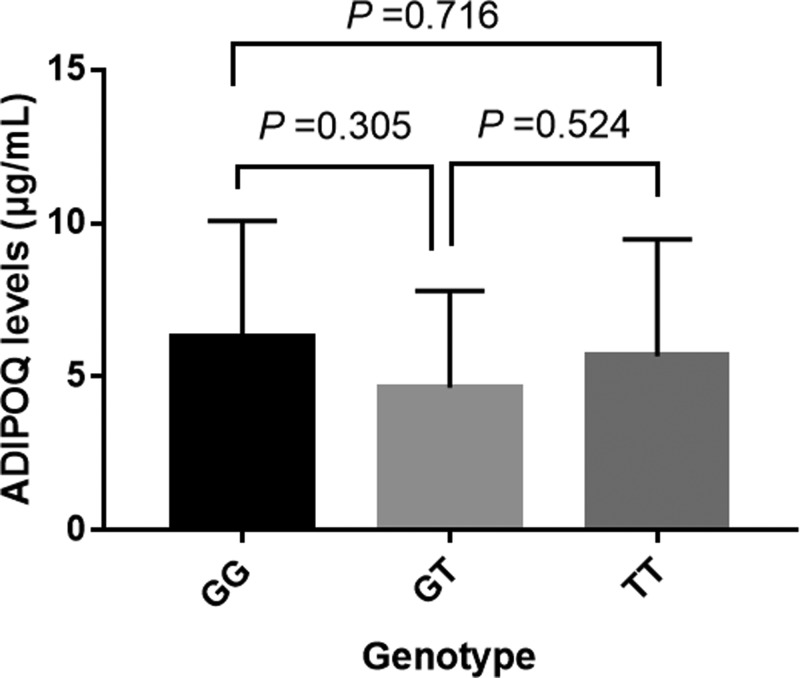
The ADIPOQ levels in different genotypes

Subgroup analyses of clinical and biochemical characteristics were also performed between groups. Significant associations were obtained in subgroups of ≥55 years (TT vs.GG: adjusted OR and 95% CI, 2.21 (1.00–4.86), *P*=0.049) and BMI < 25 kg/m^2^, but not in the analysis of sex (Table 3). The ADIPOQ level analyses in different genotypes of OA patients revealed no significant association between ADIPOQ rs1501299 polymorphism and ADIPOQ concentration (Figure 1). Furthermore, the ADIPOQ rs1501299 polymorphism did not significantly affect the K-L grades of OA patients (Table 4).

**Table 3:** The clinical and biochemical characteristics of rs1501299 polymorphism amongst two groups

Variable	rs1501299 (case/control)			GT vs. GG	TT vs. GG	TT+GT vs. GG	TT vs. GT+GG
	GG	GT	TT				
Sex							
Male	75/101	67/86	15/11	1.05 (0.68, 1.63); 0.830	1.84 (0.80, 4.23); 0.153	1.14 (0.75, 1.73); 0.544	1.80 (0.80, 4.03); 0.156
Female	99/139	93/96	22/19	1.36 (0.93, 2.00); 0.117	1.63 (0.84, 3.16); 0.153	1.40 (0.98, 2.02); 0.068	1.42 (0.75, 2.70); 0.288
Age (years)							
<55	108/148	100/107	18/18	1.28 (0.89, 1.85); 0.188	1.37 (0.68, 2.76); 0.377	1.29 (0.91, 1.84); 0.153	1.23 (0.62, 2.42); 0.556
≥55	66/92	60/75	19/12	1.12 (0.70, 1.77); 0.645	**2.21 (1.00, 4.86); 0.049**	1.27 (0.82, 1.96); 0.293	2.10 (0.98, 4.48); 0.056
BMI (kg/m^2^)							
<25	78/116	84/84	17/14	1.49 (0.98, 2.26); 0.062	1.81 (0.84, 3.87); 0.129	**1.53 (1.03, 2.29); 0.036**	1.50 (0.72, 3.13); 0.282
≥25	96/124	76/98	20/16	1.00 (0.67, 1.50); 0.993	1.62 (0.79, 3.28); 0.186	1.09 (0.74, 1.59); 0.665	1.61 (0.81, 3.21); 0.172

Bold values are statistically significant (*P*<0.05).

**Table 4:** The associations between ADIPOQ rs1501299 polymorphism and clinical characteristics of knee OA

Characteristics	Genotype distributions		TT	GT+TT	G	T
rs1501299	GG	GT				
K-L grade						
3+4/1+2	77/97	81/79	20/17	101/96	235/273	121/113
OR (95% CI); *P*-value	1.0 (reference)	1.29 (0.84, 1.99); 0.244	1.48 (0.73, 3.02); 0.279	1.33 (0.88, 2.00); 0.177	1.00 (reference)	1.24 (0.91, 1.70); 0.168

## Discussion

This case–control study showed the ADIPOQ gene rs1501299 polymorphism was significantly associated with an increased risk of knee OA. Similar associations were obtained in subgroups ≥55 years and BMI < 25 kg/m^2^.

A cross-sectional study from Finland found no relation between four ADIPOQ gene polymorphisms (including rs1501299 polymorphism) and the risk of hand OA [26]. Zhan et al. [27] from Thailand also revealed significant association between the rs1501299 genotype distribution and KL grades 2, 3, or 4, but not between ADIPOQ rs2241766 or rs1501299 polymorphism and knee OA risk. Another study from Spain indicated ADIPOQ gene rs1501299 polymorphisms were not associated with cardiovascular disease in rheumatoid arthritis patients [30], though it was not knee OA. A recent Chinese study indicated only the ADIPOQ gene rs182052 polymorphism amongst three tested SNPs was associated with OA risk [28]. Here we observed ADIPOQ gene rs1501299 polymorphism increased the risk of knee OA. To our knowledge, this is the first study to report the relation between an increased risk of OA and rs1501299 polymorphism. We hypothesized the conflicting findings amongst the above studies and the present study may be attributed to five reasons. The first potential reason was the genetic heterogeneity between Asians and Caucasians. The second one may be the clinical heterogeneity. Specifically, the study from Finland enrolled hand OA of 542 occupationally active Finnish female dentists and teachers [26], but we focussed on knee OA. Probably rs1501299 polymorphism was a specific locus for knee OA, but not hand OA. Third, the sample sizes varied amongst these studies, but small sample sizes may lead to false-positive results. We included 372 OA patients, while Jiang et al. only enrolled 172 OA cases [28]. Fourth, various occupations or body weight may also be a reason. Last but not the least, genotyping methods may also be an influencing factor. The interaction between genes and environmental factors plays a predominant role in the pathogenesis of genetic diseases. Subgroup analyses of clinical and biochemical characteristics showed significant associations in the subgroups of ≥55 years and BMI < 25 kg/m^2^, indicating these individuals were more prone to these risk factors.

Some limitations of this case–control study need to be considered. First, we only investigated one SNP of ADIPOQ gene, which called for research on other SNPs. Second, the sample size was not large enough. Third, whether ADIPOQ gene polymorphism altered the protein expression was not explored. Fourth, OA in the hip or hand joints should be further studied. Fifth, though an association in ADIPOQ gene polymorphism was observed, we did not know how this gene polymorphism influenced the knee OA development. Finally, ADIPOQ gene polymorphisms interacting with environmental factors should be explored in the future.

In conclusion, the ADIPOQ gene rs1501299 polymorphism is associated with an increased risk of knee OA in a Chinese Han population. Nevertheless, this finding should be validated by well-designed larger size case–control studies involving other populations and other joints.

## Abbreviations

ADIPOQ: adiponectin
BMI: body mass index
CI: confidence interval
HWE: Hardy–Weinberg equilibrium
K-L: Kellgren–Lawrence
OA: osteoarthritis
OR: odds ratio
SNP: single-nucleotide polymorphism

## References

[1] Brooks P.M. (2002) Impact of osteoarthritis on individuals and society: how much disability? Social consequences and health economic implications. Curr. Opin. Rheumatol. 14, 573–577 10.1097/00002281-200209000-00017 12192258

[2] Palazzo C., Nguyen C., Lefevre-Colau M.M., Rannou F. and Poiraudeau S. (2016) Risk factors and burden of osteoarthritis. Ann. Phys. Rehabil. Med. 59, 134–138 10.1016/j.rehab.2016.01.006 26904959

[3] Doherty M. (2001) Risk factors for progression of knee osteoarthritis. Lancet 358, 775–776 10.1016/S0140-6736(01)06006-8 11564477

[4] Chu I.J.H., Lim A.Y.T. and Ng C.L.W. (2018) Effects of meaningful weight loss beyond symptomatic relief in adults with knee osteoarthritis and obesity: a systematic review and meta-analysis. Obes. Rev. 19, 1597–1607 10.1111/obr.12726 30051952

[5] Szoeke C., Dennerstein L., Guthrie J., Clark M. and Cicuttini F. (2006) The relationship between prospectively assessed body weight and physical activity and prevalence of radiological knee osteoarthritis in postmenopausal women. J. Rheumatol. 33, 1835–1840 16881096

[6] Urban H. and Little C.B. (2018) The role of fat and inflammation in the pathogenesis and management of osteoarthritis. Rheumatology (Oxford) 57, iv10–iv21 10.1093/rheumatology/kex399 29444323

[7] Alrushud A.S., Rushton A.B., Kanavaki A.M. and Greig C.A. (2017) Effect of physical activity and dietary restriction interventions on weight loss and the musculoskeletal function of overweight and obese older adults with knee osteoarthritis: a systematic review and mixed method data synthesis. BMJ Open 7, e014537 10.1136/bmjopen-2016-014537 28600365 PMC5541637

[8] Leung G.J., Rainsford K.D. and Kean W.F. (2014) Osteoarthritis of the hand I: aetiology and pathogenesis, risk factors, investigation and diagnosis. J. Pharm. Pharmacol. 66, 339–346 10.1111/jphp.12196 24329488

[9] MacGregor A.J., Antoniades L., Matson M., Andrew T. and Spector T.D. (2000) The genetic contribution to radiographic hip osteoarthritis in women: results of a classic twin study. Arthritis Rheum. 43, 2410–2416 10.1002/1529-0131(200011)43:11<2410::AID-ANR6>3.0.CO;2-E 11083262

[10] MacGregor A.J., Li Q., Spector T.D. and Williams F.M. (2009) The genetic influence on radiographic osteoarthritis is site specific at the hand, hip and knee. Rheumatology (Oxford) 48, 277–280 10.1093/rheumatology/ken475 19153142 PMC2644047

[11] Valdes A.M. and Spector T.D. (2011) Genetic epidemiology of hip and knee osteoarthritis. Nat. Rev. Rheumatol. 7, 23–32 10.1038/nrrheum.2010.191 21079645

[12] Blagojevic M., Jinks C., Jeffery A. and Jordan K.P. (2010) Risk factors for onset of osteoarthritis of the knee in older adults: a systematic review and meta-analysis. Osteoarthritis Cartilage 18, 24–33 10.1016/j.joca.2009.08.010 19751691

[13] Silverwood V., Blagojevic-Bucknall M., Jinks C., Jordan J.L., Protheroe J. and Jordan K.P. (2015) Current evidence on risk factors for knee osteoarthritis in older adults: a systematic review and meta-analysis. Osteoarthritis Cartilage 23, 507–515 10.1016/j.joca.2014.11.019 25447976

[14] Scherer P.E. (2006) Adipose tissue: from lipid storage compartment to endocrine organ. Diabetes 55, 1537–1545 10.2337/db06-0263 16731815

[15] Kitahara K., Kusunoki N., Kakiuchi T., Suguro T. and Kawai S. (2009) Adiponectin stimulates IL-8 production by rheumatoid synovial fibroblasts. Biochem. Biophys. Res. Commun. 378, 218–223 10.1016/j.bbrc.2008.11.017 19013427

[16] Kusunoki N., Kitahara K., Kojima F., Tanaka N., Kaneko K., Endo H. (2010) Adiponectin stimulates prostaglandin E(2) production in rheumatoid arthritis synovial fibroblasts. Arthritis Rheum. 62, 1641–1649 10.1002/art.27450 20222108

[17] Tang C.H., Chiu Y.C., Tan T.W., Yang R.S. and Fu W.M. (2007) Adiponectin enhances IL-6 production in human synovial fibroblast via an AdipoR1 receptor, AMPK, p38, and NF-kappa B pathway. J. Immunol. 179, 5483–5492 10.4049/jimmunol.179.8.5483 17911635

[18] Conde J., Scotece M., Gomez R., Lopez V., Gomez-Reino J.J. and Gualillo O. (2011) Adipokines and osteoarthritis: novel molecules involved in the pathogenesis and progression of disease. Arthritis 2011, 203901 10.1155/2011/203901 22046513 PMC3200120

[19] Giles J.T., van der Heijde D.M. and Bathon J.M. (2011) Association of circulating adiponectin levels with progression of radiographic joint destruction in rheumatoid arthritis. Ann. Rheum. Dis. 70, 1562–1568 10.1136/ard.2011.150813 21571734 PMC3543946

[20] Klein-Wieringa I.R., van der Linden M.P., Knevel R., Kwekkeboom J.C., van Beelen E., Huizinga T.W. (2011) Baseline serum adipokine levels predict radiographic progression in early rheumatoid arthritis. Arthritis Rheum. 63, 2567–2574 10.1002/art.30449 21567382

[21] Koskinen A., Juslin S., Nieminen R., Moilanen T., Vuolteenaho K. and Moilanen E. (2011) Adiponectin associates with markers of cartilage degradation in osteoarthritis and induces production of proinflammatory and catabolic factors through mitogen-activated protein kinase pathways. Arthritis Res. Ther. 13, R184 10.1186/ar3512 22077999 PMC3334633

[22] Gross J.B., Guillaume C., Gegout-Pottie P., Mainard D. and Presle N. (2014) Synovial fluid levels of adipokines in osteoarthritis: association with local factors of inflammation and cartilage maintenance. Biomed. Mater. Eng. 24, 17–25 24928914 10.3233/BME-140970

[23] King L.K., Henneicke H., Seibel M.J., March L. and Anandacoomarasmy A. (2015) Association of adipokines and joint biomarkers with cartilage-modifying effects of weight loss in obese subjects. Osteoarthritis Cartilage 23, 397–404 10.1016/j.joca.2014.11.020 25481288

[24] Fioravanti A., Cheleschi S., De Palma A., Addimanda O., Mancarella L., Pignotti E. (2018) Can adipokines serum levels be used as biomarkers of hand osteoarthritis? Biomarkers 23, 265–270 10.1080/1354750X.2017.1401665 29105498

[25] Tang Q., Hu Z.C., Shen L.Y., Shang P., Xu H.Z. and Liu H.X. (2018) Association of osteoarthritis and circulating adiponectin levels: a systematic review and meta-analysis. Lipids Health Dis. 17, 189 10.1186/s12944-018-0838-x 30115130 PMC6097292

[26] Hamalainen S., Solovieva S., Vehmas T., Hirvonen A. and Leino-Arjas P. (2018) Adipokine genes and radiographic hand osteoarthritis in Finnish women: a cross-sectional study. Scand. J. Rheumatol. 47, 71–78 10.1080/03009742.2017.1314000 28812414

[27] Zhan D., Thumtecho S., Tanavalee A., Yuktanandana P., Anomasiri W. and Honsawek S. (2017) Association of adiponectin gene polymorphisms with knee osteoarthritis. World J. Orthop. 8, 719–725 10.5312/wjo.v8.i9.719 28979856 PMC5605358

[28] Jiang L., Zhu X., Rong J., Xing B., Wang S., Liu A. (2018) Obesity, osteoarthritis and genetic risk: The rs182052 polymorphism in the ADIPOQ gene is potentially associated with risk of knee osteoarthritis. Bone Joint Res. 7, 494–500 10.1302/2046-3758.77.BJR-2017-0274.R1 30123499 PMC6076358

[29] Kellgren J.H., Lawrence J.S. and Bier F. (1963) Genetic factors in generalized osteo-arthrosis. Ann. Rheum. Dis. 22, 237–255 10.1136/ard.22.4.237 14043587 PMC1030831

[30] Rodriguez-Rodriguez L., Garcia-Bermudez M., Gonzalez-Juanatey C., Vazquez-Rodriguez T.R., Miranda-Filloy J.A., Fernandez-Gutierrez B. (2011) Lack of association between ADIPOQ rs266729 and ADIPOQ rs1501299 polymorphisms and cardiovascular disease in rheumatoid arthritis patients. Tissue Antigens 77, 74–78 10.1111/j.1399-0039.2010.01580.x 21073447

